# Why Is 10 Past 10 the Default Setting for Clocks and Watches in Advertisements? A Psychological Experiment

**DOI:** 10.3389/fpsyg.2017.01410

**Published:** 2017-08-23

**Authors:** Ahmed A. Karim, Britta Lützenkirchen, Eman Khedr, Radwa Khalil

**Affiliations:** ^1^Department of Prevention and Health Psychology, SRH Fernhochschule – The Mobile University Riedlingen, Germany; ^2^Department of Psychiatry and Psychotherapy, University of Tübingen Tübingen, Germany; ^3^Department of Neuropsychology, Jacobs University Bremen, Germany; ^4^Department of Neuropsychiatry, Assiut University Hospital Assiut, Egypt; ^5^Center for Molecular and Behavioral Neuroscience, Behavioral and Neural Sciences Graduate Program, Rutgers University, Newark NJ, United States

**Keywords:** face perception, emotion, subliminal perception, neuromarketing, product design

## Abstract

Have you ever noticed that in watch advertisements the time is usually set at 10:10? The reasons and psychological effects of this default time setting are elusive. In Experiment 1, we hypothesized that watches showing a time setting resembling a smiling face (10:10) would enhance emotional valence and intention to buy compared to a neutral time setting (11:30), whereas a time setting resembling a sad face (8:20) would have the opposite effect. Moreover, we investigated a possible interaction effect with the gender of the participants. In Experiment 2, we directly tested the hypotheses that watches set at 10:10 resemble a smiling face, whereas watches set at 8:20 resemble a sad face. The data of the first experiment reveal that watches set at 10:10 showed a significant positive effect on the emotion of the observer and the intention to buy. However, watches set at 8:20 did not show any effect on the emotion or the intention to buy. Moreover, watches set at 10:10 induced in women significantly stronger ratings of pleasure than in men. The data of the second experiment show that participants consistently perceive high resemblance between watches set at 10:10 and a smiling face as well as high resemblance between watches set at 8:20 and a sad face. This study provides for the first time empirical evidence for the notion that using watches with a time setting resembling a smiling face (like 10:10) can positively affect the emotional response of the observers and their evaluation of a seen watch, even though they are not aware of the fact that the shown time setting is inducing this effect. Practical implications of the observed findings and alternative explanations are discussed.

## Introduction

Recent brain imaging studies suggest that subliminal stimuli can alter behavior, via non-conscious processes (Eimer and Schlaghecken, 2003; Muscarella et al., 2013). Masked facial expressions have been shown to influence the activation of the amygdala by subsequent visible faces (Whalen et al., 1998; Kim et al., 2010, 2016) thus demonstrating that some aspects of face processing can occur without conscious awareness. Similarly, Morris et al. (2007) investigated brain activity evoked by masked faces which were not consciously perceived by subjects and found a response from the fusiform gyrus (FFG) capable of discriminating faces from other objects even when all trials occur outside of conscious awareness. Further neuroimaging studies showed that in comparison to other objects seeing faces activates emotional processes and correlated neural networks which increases the storing of the presented information (Eimer, 2000; Wiese et al., 2014). Suzuki and Cavanagh (1995) have shown that the recognition of face similar structures occurs faster than other objects. Moreover, several studies demonstrated the preference of newborn babies for stimuli that contain the basic configuration of a face, whereas the mechanisms that underlie this preference are still the focus of debate (for a review see Johnson, 2005).

These results suggest that within the context of marketing the use of faces, which can modulate emotions, could be profitable (Silberstein and Nield, 2012). Murphy and Zajonc (1993) have shown that a stimulus is experienced more positively if there was a smiling face shown before. Thus, their data reveal that it is possible to influence human judgment through the priming presentation of faces. Affective primacy was first suggested by a mere exposure experiment conducted by Kunst-Wilson and Zajonc (1980), in which they showed that repeated exposure to a stimulus creates a preference for it, even if the stimuli are presented subliminally. In their experiment ideographs were first presented under degraded viewing conditions. Participants still preferred the stimuli they have seen before to new ones but were not able to consciously differentiate between the old and the new ones. Moreover, Murphy and Zajonc (1993) found that extremely brief exposure to an effective prime (facial expressions) could bias subjects’ judgment of a neutral stimulus (Chinese ideograms). This effect, which they call affective priming, challenges the cognitive appraisal viewpoint (Lazarus, 1982), which maintains that affect cannot emerge without prior cognitive mediation (for a review on the debate between the affective primacy hypothesis and the cognitive appraisal viewpoint (see e.g., Russ, 2003; Payne et al., 2010; Goller et al., 2017). Recent neuroimaging studies demonstrate that subliminal stimuli and masked facial expressions can alter behavior via non-conscious processes activating the FFG and subcortical regions like the amygdala (Whalen et al., 1998; Johnson, 2005; Morris et al., 2007; Prete et al., 2015).

Intriguingly, since the 1950s in watch advertisements the time has commonly been set at 10:10 (Newman, 2008), assuming that this default time setting will positively affect consumers, because it resembles a smiling face, although consumers neither consciously notice this default time setting nor are they aware of this intended resemblance of a smiling face. A search in galleries such as Adclassix.com indicates that 10:10 was not always the norm. In the 1920s and 1930s, watches were almost always set at 8:20, which had the aesthetic advantage of being symmetrical and not overshadowing logos but resembled a sad face.

However, the assumption that time settings resembling different facial expressions affect consumers has, to our knowledge, never been experimentally tested.

The aim of this study was therefore to investigate for the first time the effects of a time setting (10:10) resembling certain features of a happy facial expression compared with a time setting (8:20) resembling certain features of a sad facial expression and a neutral time condition (11:30) on the emotional response and the intention to buy of consumers. Moreover, we intended to investigate the effects of gender on the perception of time settings resembling smiling and sad facial expressions, since several studies have shown that women are superior to men at recognizing facial expressions of emotion (Hall, 1984; Babchuk et al., 1985; Hampson et al., 2006). Hampson et al. (2006) evaluated whether the expression of sex difference is influenced by the valence of the emotional signal (positive or negative). Their results showed that women were faster than men at recognizing both positive *and* negative emotions from facial cues.

Results from meta analyses show that there is a female advantage in recognizing facially expressed emotions even though the mean effect size seems to be rather small (Hall, 1978; Hall et al., 2000). Remarkably, gender differences become much more apparent when facial stimuli are presented at the edge of conscious awareness (Hall and Matsumoto, 2004; Hoffmann et al., 2010). Thus, women appear to recognize facial emotions better than men in particular under conditions of minimal stimulus information, i.e., when facial expression is shown for less than a second (Donges et al., 2012). Neuroimaging studies also show gender effects in neurofunctional mechanisms of emotion and cognition in some brain regions (e.g., Azim et al., 2005; Hofer et al., 2006; Schulte-Rüther et al., 2008). Hofer et al. (2006) have reported increased activation in women compared with men in the right posterior cingulate, the left putamen and the left cerebellum during positive mood induction. Schulte-Rüther et al. (2008) have demonstrated that females show increased activation of the right inferior frontal cortex when evaluating the emotional states expressed by faces. These studies provide valuable insights into the neural correlates of gender differences in recognizing and evaluating facial expressions.

In this study, two experiments were conducted to investigate the effects of watches showing different time settings resembling certain features of happy or sad facial expression. In the first experiment, we hypothesized that watches showing a time setting resembling a smiling face (10:10) would enhance emotional valence and intention to buy compared to a neutral time setting (11:30), whereas a time setting resembling a sad face (8:20) would have the opposite effect. Moreover, we intended to test a possible interaction effect with the gender of the participants. In the second experiment, we directly tested the hypotheses that watches set 10:10 resemble a smiling face, whereas watches set at 8:20 resemble a sad face.

## Materials and Methods

### Subjects

Forty-six subjects (20 men and 26 women) participated in the first experiment. Their age ranged between 20 and 45 years; the mean age ± SD was 29.0 ± 6.3. Twenty-three subjects (11 men and 12 women) participated in the second experiment. Their age ranged between 22 and 51 years; the mean age ± SD was 34.4 ± 10. According to Rosner (1995) if we assume a middle effect size between 0.4 and 0.6 and a power estimate (1-β) of 0.8 we would need a sample size (for within subject comparisons under different conditions) between *N* = 22 and *N* = 49.

All subjects provided written informed consent, and were naive about the hypotheses and the aim of the study. The study was conducted in strict accordance with the local ethics policies and all procedures involved were in accordance with the latest version of the Declaration of Helsinki (WMA, 2013). The study was approved by the Internal Review Board of the SRH University in Riedlingen, Germany.

### Experimental Design

#### Experiment 1

**FIGURE 1:**
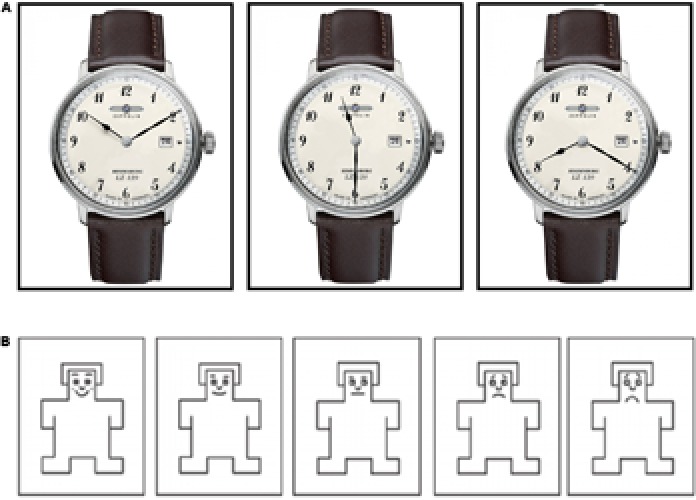
Examples of the stimuli used in the first experiment. **(A)** Depicts one of the used watches with the three different time settings (10:10, 11:30, and 08:20). **(B)** Shows the pleasure scale from the Self-Assessment Manikin (SAM) of Bradley and Lang (1994). The most positive face was coded with number 5 while the most negative face was coded with number 1.

Stimuli were generated from color digital photographs of twenty different watches with a white background. Each watch was photographed with three different time settings: (A) 10:10; (B) 11:30 and (C) 8:20 resulting in sixty pictures. **Figure 1A** depicts one of the used watches with the three different time settings. Subjects were told that they will see different watches and that they were to rate their perceived emotional response on the pleasure scale of the Self-Assessment Manikin (SAM) of Bradley and Lang (1994) while seeing one of these watches. Afterward, they were asked to rate their intention to buy such a watch. The survey was performed in an experimental room. Stimuli were presented on 400 × 400-pixel array on a 21″ Samsung computer monitor. The watch images had a height of 10 cm and a width of 3–5 cm. Images were shown in random order [(1) instruction: “Please look at this watch”]. When the participant pressed the mouse button the image of the watch appeared again, but this time with the SAM pleasure scale below the image [(2) Instruction: “What do you feel, when you look at this watch? Please rate your emotional response on the following scale”]. Ratings were obtained by a mouse-controlled cursor. Then, the watch image appeared for the third time, but this time with the intention to buy scale below the image. The (3) instruction was: “How likely is it, that you would buy this watch?” Please choose a value between -10 (on no account) to +10 (on all accounts). This procedure was then repeated for all 60 watch images. The time for evaluating the watches on the two scales wasn’t restricted. The actual duration averaged ± SD 19.0 min ± 7.2.

At the end of the task, subjects were asked about their age and gender. Finally, their awareness of the study purpose and of the shown time setting was asked with open-ended questions (“Did you notice anything special concerning the watches? If yes, what did you notice? Did you notice anything special concerning the time setting? If yes, what did you notice? Did you notice anything special concerning the wristbands? If yes, what did you notice?”)

None of the subjects noticed that watches were presented at three specific time settings, thus all subjects were included in the final analyses.

#### Experiment 2

**FIGURE 2:**
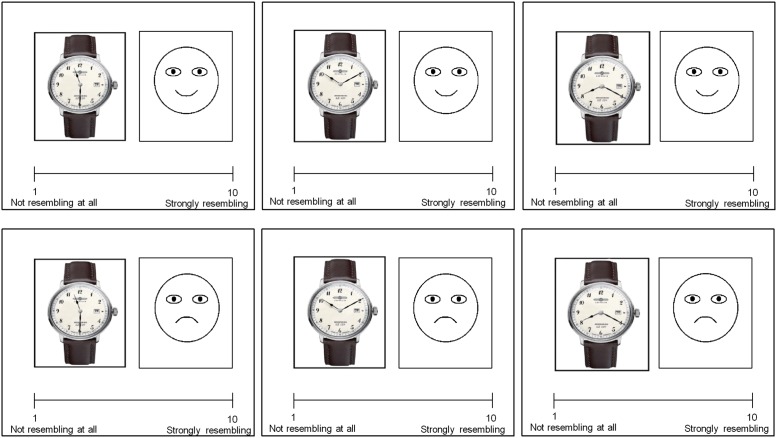
Examples of the stimuli used in the second experiment. Subjects were asked to compare watches with facial emotional expressions and to rate the resemblance between the facial emotional expression (smiling versus sad face) and the shown time setting (10:10, 11:30, and 08:20) on a scale from 1 (not resembling at all) to 10 (strongly resembling). The combination of time setting and facial emotional expression was randomized.

In this experiment, subjects obtained the following instruction: “You will see watches with different time settings. Please rate on a scale from 1 (not resembling at all) to 10 (strongly resembling) how far these time settings resemble facial emotional expressions.” The stimuli consisted of four watches, each one photographed with three different time settings (10:10; 11:30 and 8:20) resulting in a total number of twelve pictures. The features of these stimuli were identical to the first experiment. Subjects were shown in randomized order one of the watches with one of the three-time settings next to a pictogram of a smiling or a sad face and were asked to rate the resemblance between them on a scale from 1 (not resembling at all) to 10 (strongly resembling). **Figure 2** shows an example of the used stimuli. The survey in this experiment was performed under the same conditions as in the first experiment. The time for evaluating the watches wasn’t restricted. At the end of the task, subjects were asked about their age and gender. The actual duration of this experiment averaged ± SD 6.2 min ± 2.3.

#### Measurement of the Emotional Response in Experiment 1

In order to measure the emotional response to the three different time settings, the pleasure scale of the SAM of Bradley and Lang (1994) was used. The pleasure scale ranges from a smiling, happy figure to a frowning, unhappy figure. The subjects had to rate their pleasure while seeing the different watches by choosing one of the pictograms shown in **Figure 1B**.

Self-assessment manikin is a well validated non-verbal pictorial assessment technique that directly measures the pleasure, arousal, and dominance associated with a person’s affective reaction to a wide variety of stimuli. Bradley and Lang (1994) argue that SAM may elicit more consistent judgments than a verbal scale (semantic differential) because the SAM figure itself is human-like. The translation of personal experience to numerical values, such as in Likert scales can be sometimes problematic: While some might assume that the numbers represent equidistant categories for judging emotional response, others might interpret the same scale as ordinal (for an in-depth discussion on this issue see, e.g., Obaid et al., 2015).

#### Measurement of the Intention to Buy in Experiment 1

Subjects had to rate their intention to buy the shown watches on a scale ranging from -10 (on no account) to +10 (on all accounts).

## Results

### Experiment 1

#### Effects of the Time Settings on the Emotional Response

**FIGURE 3:**
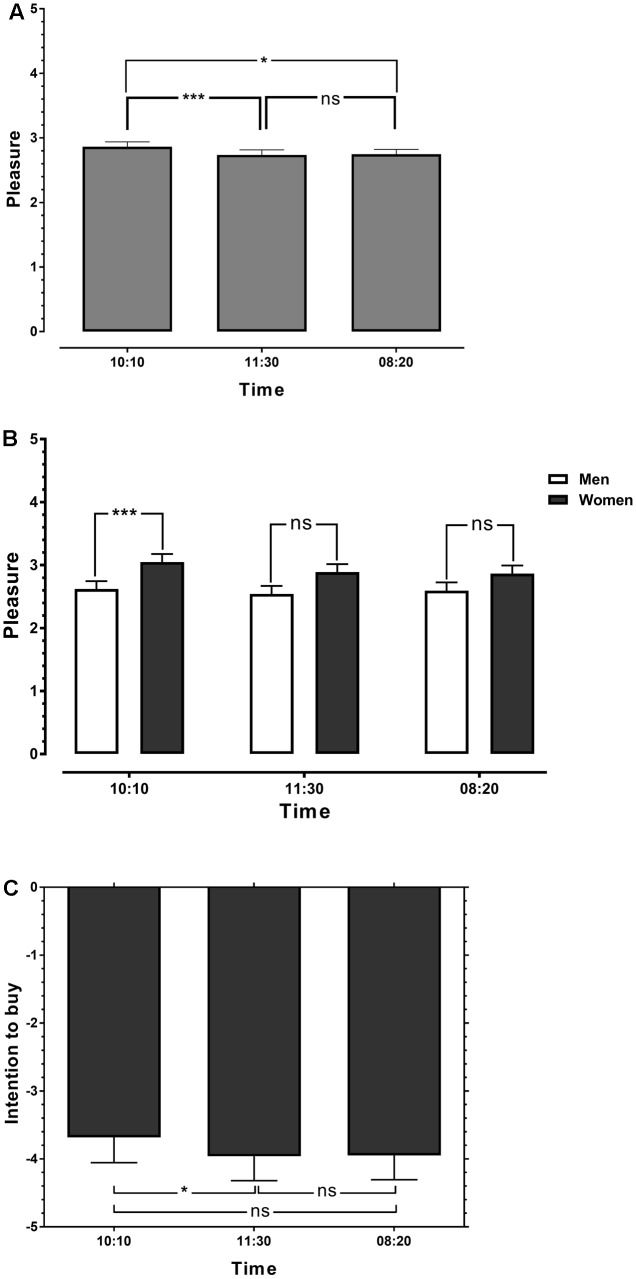
Effects of time setting (10:10, 11:30, and 08:20) on the emotional responses of the subjects **(A)**. **(B)** Demonstrates the modulating effect of gender. **(C)** Shows the effect of time setting on the intention to buy. Error bars denote the standard error of the mean (SEM). ^∗^*P* < 0.05; ^∗∗∗^*P* < 0.001.

A one-way repeated measures analyses of variance (ANOVA) with TIME _(10:10/11:30/8:20)_ as within-subject factor and PLEASURE as dependent variable revealed a significant main effect for TIME (*F*_2,45_ = 9.837, *P* < 0.001, η^2^ = 0.179). Bonferroni corrected *post hoc* paired *t*-tests showed that watches set at 10:10 caused significantly higher pleasure than watches set at 11:30 (*t*_45_ = 3.817, *P* < 0.001) and watches set at 8:20 (*t*_45_ = 3.180, *P* < 0.005). However, no significant difference was found between watches set at 11:30 and watches set at 8:20 (*t*_45_ = 0.396, *P* = 0.719; see **Figure 3A**).

Furthermore, a repeated measures ANOVA with GENDER _(male/female)_ as between-subject factor and TIME _(10:10/11:30/8:20)_ as within-subject factor revealed a significant interaction between TIME and GENDER (*F*_2,44_ = 3.413, *P* < 0.05, η^2^= 0.072). Bonferroni corrected *post hoc t*-tests revealed that women experienced significantly higher pleasure seeing watches set at 10:10 than men (*t*_45_ = 3.01, *P* < 0.01), whereas no significant differences between men and women were found for the other time settings (see **Figure 3B**).

#### Effects of the Time Settings on the Intention to Buy

A one-way repeated measures ANOVA with TIME _(10:10/11:30/8:20)_ as within-subject factor and INTENTION TO BUY as dependent variable revealed a significant main effect for TIME (*F*_2,45_ = 3.608, *P* = 0.031, η^2^= 0.074). Bonferroni corrected *post hoc* paired *t*-tests showed that watches set at 10:10 caused a significantly higher intention to buy than watches set at 11:30 (*t*_45_ = 2.430, *P* < 0.05). Watches set at 10:10 showed also the tendency to induce a higher intention to buy than watches set at 8:20. However, this effect missed significance (*t*_45_ = 2.009, *P* = 0.051). No significant difference was found between watches set at 11:30 and watches set at 8:20 (*t*_45_ = 0.117, *P* = 0.907; see **Figure 3C**).

A repeated measures ANOVA with GENDER _(male/female)_ as between-subject factor and TIME _(10:10/11:30/8:20)_ as within-subject factor revealed no significant Interaction between TIME and GENDER (*F*_2,44_ = 0.635, *P* = 0.532, η^2^= 0.014) concerning the intention to buy.

### Experiment 2

#### Effects of the Time Setting on the Resemblance to a Smiling Face

**FIGURE 4:**
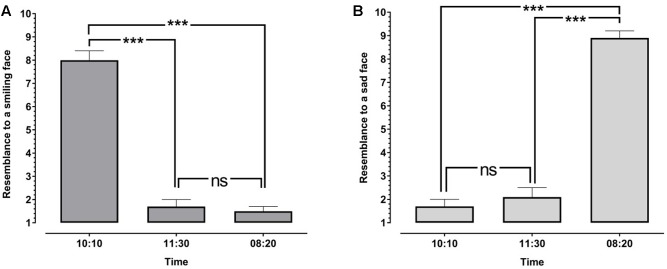
Effects of time setting (10:10, 11:30, and 08:20) on the perceived resemblance to a smiling face **(A)** and to a sad face **(B)**. Error bars denote the standard error of the mean (SEM). ^∗^*P* < 0.05; ^∗∗∗^*P* < 0.001.

A one-way repeated measures ANOVA with TIME _(10:10/11:30/8:20)_ as within-subject factor and RESEMBLANCE TO A SMILING FACE as dependent variable revealed a highly significant main effect for TIME (*F*_2,22_ = 155.249, *P* < 0.001, η^2^= 0.876). Bonferroni corrected *post hoc* paired *t*-tests showed that watches set at 10:10 are perceived as significantly resembling a smiling face much more than watches set at 11:30 (*t*_22_ = 14.341, *P* < 0.001) or watches set at 8:20 (*t*_22_ = 14.689, *P* < 0.001). No significant difference concerning the resemblance to a smiling face was found between watches set at 11:30 and watches set at 8:20 (*t*_22_ = 0.699, *P* = 0.492; see **Figure 4A**).

#### Effects of the Time Setting on the Resemblance to a Sad Face

A one-way repeated measures ANOVA with TIME _(10:10/11:30/8:20)_ as within-subject factor and RESEMBLANCE TO A SAD FACE as dependent variable revealed a highly significant main effect for TIME (*F*_2,22_ = 175.980, *P* < 0.001, η^2^= 0.889). Bonferroni corrected *post hoc* paired *t*-tests showed that watches set at 08:20 are perceived as significantly resembling a sad face much more than watches set at 11:30 (*t*_22_ = 17.285, *P* < 0.001) or watches set at 10:10 (*t*_22_ = 19.384, *P* < 0.001). No significant difference concerning the resemblance to sad faces was found between watches set at 11:30 and watches set at 10:10 (*t*_22_ = 0.762, *P* = 0.454; see **Figure 4B**).

## Discussion

This study provides for the first time empirical evidence for the notion that using watches with a time setting resembling a smiling face (like 10:10) can positively affect the emotional response of the observers and their evaluation of a seen watch, even though they are not aware of the fact that the shown time setting is inducing this effect. In the first experiment participants were shown consecutively twenty different watches, each with one of the following time settings: (A) 10:10, which is supposed to resemble a smiling face; (B) 8:20, which is supposed to resemble a sad face, and (C) 11:30 as a neutral time setting condition. Our findings show that watches set at 10:10 induce significantly stronger feelings of pleasure compared with the other time settings. This effect is intriguing because since the 1950s in watch advertisements the time has commonly been set at 10:10, assuming that this time setting will positively affect costumers, although in earlier decades the default time setting was 8:20. Both time settings, 10:10 *and* 8:20, have the aesthetic advantage of being symmetrical and not overshadowing the logo. However, our findings reveal that watches set at 8:20 did not significantly affect feelings of pleasure, neither positively nor negatively.

Moreover, watches set at 10:10 induced in women significantly stronger ratings of pleasure than in men. This effect seems to be in line with previous studies showing that women are superior to men at recognizing facial expressions of emotion and empathizing with them (Hall, 1984; Babchuk et al., 1985; Hampson et al., 2006). Neuroimaging studies also reveal gender effects in the neurofunctional mechanisms of emotion and cognition in some brain regions (e.g., Azim et al., 2005; Hofer et al., 2006; Schulte-Rüther et al., 2008).

The observed positive effect of watches set at 10:10 was, however, not strong enough to allure the consumers to buy a watch. In fact, on average the consumers decided rather not to buy any of the shown watches. Still, watches set at 10:10 significantly *reduced* the reluctance to buy a watch as shown in **Figure 3C**. In contrast, the time setting 8:20 didn’t have a significant effect on the intention to buy compared with the neutral condition (time setting at 11:30).

Our second experiment shows that when participants explicitly compare the three different time settings (10:10; 8:20 and 11:30) with pictograms of a smiling and a sad face, they consistently perceive high resemblance between watches set at 10:10 and a smiling face as well as high resemblance between watches set at 8:20 and a sad face.

Yet, several limitations of this study have to be discussed. The resemblance of watches set at 10:10 with a smiling face might not be the only possible reason for the observed positive effects on emotional valence. One might argue, as an alternative explanation, that lines and shapes (e.g., watch hands) that point up are generally perceived more positively than those pointing down, similar to a thumbs up sign. Previous studies have demonstrated that simple geometric shapes may convey emotional meaning using various experimental paradigms (Aronoff et al., 1988; Watson et al., 2012; Wang and Zhang, 2016; Salgado-Montejo et al., 2017). For instance, according to LoBue (2014), simple visual stimuli that are not associated with a facial gesture can nevertheless still be associated with threat; specifically, lines resembling a snake. Nevertheless, there is cumulating evidence that even simple lines and shapes would be associated with emotions if they present face-like features (see e.g., Aronoff et al., 1988; Watson et al., 2012; Salgado-Montejo et al., 2017). Our findings are in agreement with a study of Salgado-Montejo et al. (2015) in which they examined the possibility that subtle face-like features (a smile- or a frown-like line) on a product can influence evaluations of and preferences for that product. In their study participants viewed four variants (concave [smile-like] line, convex [frown-like] line, straight line, line absent) of three different products (tea, shampoo, juice) and evaluated the products on visual analog scales and completed a forced choice decision task. Their results revealed a general tendency across scales, products, and countries for the participants to rate products more positively and to choose products more frequently when they displayed a concave line relative to a convex line. In a recent study Salgado-Montejo et al. (2017) investigated whether lines and shapes that present face-like features would be associated with emotions. They reported that participants found it easiest to associate the concave line with the word “happy” and the convex line with the word “sad.”

Moreover, our findings are also in line with recent neuroimaging studies demonstrating that subliminal stimuli and masked facial expressions can alter behavior via non-conscious processes activating the FFG and subcortical regions like the amygdala (Whalen et al., 1998; Johnson, 2005; Morris et al., 2007; Prete et al., 2015). Future neuroimaging studies should, therefore, investigate if a time setting (10:10) resembling a smiling face also activates the FFG and/or the amygdala compared with other time settings.

In this study, we only investigated the effects of three distinct time settings (10:10; 8:20 and 11:30). Future studies could investigate psychophysical thresholds gradually varying the time setting and measuring the corresponding psychological and emotional responses. Moreover, we did not measure the participants’ preferences for specific watch brands and styles, which might explain why the participants in our study were reluctant to buy any of the shown watches. Future studies could, therefore, show each participant an individualized set of watches based on his/her preferences and investigate in these watches the effects of different time settings on their emotional responses and judgments.

A further possible limitation in our study could be seen in the utilization of the SAM scale from Bradley and Lang (1994) which also uses facial expressions. It is thus possible that participants - without explicitly noting this – rated the similarity of clock faces to facial expressions instead of really referring to the abstract category of pleasure in Experiment 1. Although the SAM scales provide several methodological advances compared to a verbal scale (for an in-depth discussion on this issue see Bradley and Lang, 1994; Obaid et al., 2015), it would be interesting to replicate our study with a purely text-based rating scale of valence and investigate if our main findings stay consistent.

One might also argue that we simply like what we are familiar with, or have repeatedly been exposed to Carr et al. (2017). Nowadays 10:10 is the default time setting we are usually exposed to in advertisements; hence this could explain why we like this specific time setting. However, this assumption cannot explain, why since the 1950s in watch advertisements the time setting has actually changed from 8:20 to 10:10 (cf. Newman, 2008). Nevertheless, it would be tempting to replicate our study in an ethnic culture or population group with no or seldom exposure to watch advertisements and investigate if they would still prefer watches set at 10:10 over watches set at 8:20 or 11:30.

Concerning marketing strategies, it would be interesting to further investigate the effects of integrating features resembling smiling faces into products as shown by Salgado-Montejo et al. (2015) and evaluate the modulating effects of gender, age and other sociocultural variables. Moreover, ethical challenges in using unconscious processes and neuroscientific findings in advertisement and marketing should be discussed (cf. Farah, 2002; Karim, 2010).

Combing fMRI with the Facial Action Coding System (FACS, Ekman et al., 2002) we have previously shown the somatotopic organization of emotional facial expressions in the brain and investigated the pathophysiology of deficits in recognizing facial emotional expressions and theory of mind skills in neuropsychiatric disorders (Krippl and Karim, 2011; Krippl et al., 2015; see also Baron-Cohen et al., 2009; Morris et al., 2009; Demenescu et al., 2010; Feuerriegel et al., 2015). Based on these clinical findings we assume that the observed effects in this study will be absent or at least hindered in patients with Prosopagnosia (Hwang et al., 2012) and Autism (Baron-Cohen et al., 2009; Krippl and Karim, 2011; Feuerriegel et al., 2015). Moreover, it would be interesting to investigate the effects of time settings resembling facial emotional expressions in further neuropsychiatric patients with theory of mind deficits.

## Author Contributions

All authors listed have made a substantial, direct and intellectual contribution to the work, and approved it for publication.

## Conflict of Interest Statement

The authors declare that the research was conducted in the absence of any commercial or financial relationships that could be construed as a potential conflict of interest.
